# Cutting-edge deep-learning based tools for metagenomic research

**DOI:** 10.1093/nsr/nwaf056

**Published:** 2025-02-19

**Authors:** Eli Levy Karin, Martin Steinegger

**Affiliations:** ELKMO, Copenhagen 2720, Denmark; School of Biological Sciences, Seoul National University, Seoul 08826, Republic of Korea; Interdisciplinary Program in Bioinformatics, Seoul National University, Seoul 08826, Republic of Korea; Artificial Intelligence Institute, Seoul National University, Seoul 08826, Republic of Korea; Institute of Molecular Biology and Genetics, Seoul National University, Seoul 08826, Republic of Korea

**Keywords:** metagenomics, deep learning, microbial, structure prediction, function annotation

## Abstract

Recent years have seen incredible progress in the development of deep-learning (DL) tools for the analysis of biological data, with the most prominent example being AlphaFold2 for accurate protein structure prediction. DL-based tools are especially useful for identifying patterns and connections within sparsely labeled datasets. This makes them essential for the analysis of metagenomic data, which is mostly unannotated and bears little sequence similarity to known genes and proteins. In this review, we chose to present 12 tools which we deem as offering novel capabilities for metagenomic analysis by utilizing interesting DL techniques. This review is thus intended to be a solid starting point for any data scientist looking to apply advanced methods to explore metagenomic datasets. For each DL-based tool, we present its computational principles, followed by relevant examples of its application where possible and a note on its limitations.

## INTRODUCTION

In metagenomics, genetic material is collected directly from environmental samples, allowing for the study of organisms in their natural environments. While metagenomics holds great potential for expanding our understanding of biological diversity, it also poses considerable analysis challenges. Metagenomic datasets are often vast, complex, and mostly unlabeled and unannotated [1]. Furthermore, a substantial portion of metagenomic data bears little sequence similarity to known genes and proteins, making traditional analysis methods insufficient.

To address these challenges, deep learning (DL), a specialized branch of machine learning, has become an invaluable approach. DL employs multi-layered (‘deep’) neural networks (DNNs) to decipher intricate patterns within large and complex datasets [2]. Its roots date back to the 1940s with the advent of the perceptron model, but it wasn't until the 1980s and 1990s that the development of backpropagation and the increase in computational power allowed for the training of networks with multiple layers. In the field of biology, DL has been used since the early 2000s, with applications ranging from predicting protein structures to analyzing genetic sequences (reviewed in [3]).

The most prominent DL achievement in biology is undoubtedly the development of AlphaFold2 [4]. This tool has revolutionized protein structure prediction, achieving unprecedented accuracy *on par* with experiments in the Critical Assessment of protein Structure Prediction (CASP14) competition [5]. Other notable applications include the use of DL for gene expression and regulation [6,7], drug discovery [8] and disease diagnosis [9,10].

**Figure 1.**
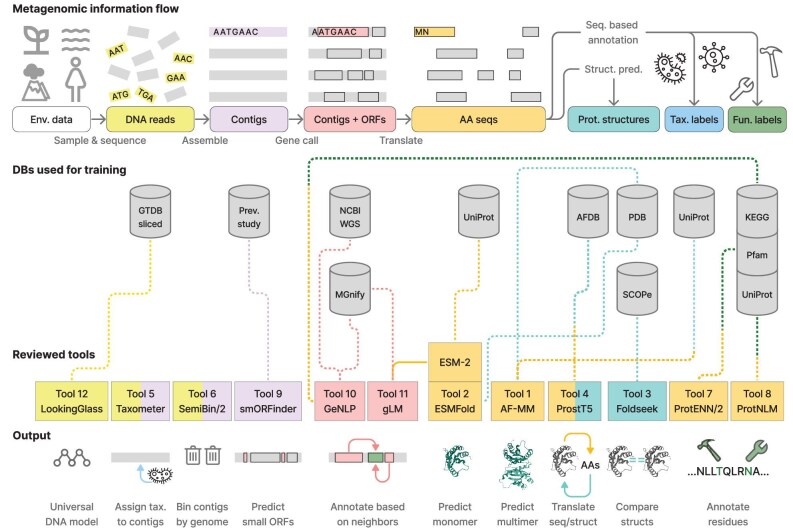
DL-based tools for metagenomics in this review. A simplified and linearized information flow in a typical metagenomic study results in various data types, such as DNA reads (yellow) and identified Open Reading Frames (ORFs) in assembled contigs (pink). Amino-acid sequences (AA seqs, orange) translated from ORFs can be used in various ways for protein structure prediction (turquoise), taxonomic (blue), and functional (green) annotation. Each tool (Table 1: Links to software and models) is colored by the type of data it takes as input and is connected by a dashed line to the databases, which were used for its training (Table 2: Details about the databases). The color of the line indicates the type of information obtained from the database during training. Tools 2 and 11 use the protein language model ESM-2 and it is thus depicted as connected to them. Tools 5 and 6 train the network on-the-fly with each input sample(s) and are thus not depicted as connected to any database. The tools are organized in two sections in this review by the task they perform, which is reflected in their output (bottom): ‘I. Protein structure prediction and analysis’ (tools 1–4) and ‘II. Contig taxonomy and binning, protein coding gene calling and functional annotation’ (tools 5–12).

While there are numerous DL-based tools for metagenomics, in this review we chose to focus on 12 of them that together describe the current forefront of using DL techniques in the field. We thus encourage readers to further explore the literature (see reviews [11–15]) about any specific inference task or DL approach of their interest. Focusing on fewer tools allowed us to accompany the tools’ presentation with biological use cases, which we hope can inspire readers; relate the tools to one another, and where possible, in the limitations section of each tool, propose alternative DL- and non–DL-based tools for performing the same computational task. This review is divided into two sections, according to the task performed by the selected tools (Fig. 1). The links to the selected tools are provided in Table 1 and Table 2 provides information about the databases used for their training. These databases serve not only as the foundation for the tools but also as key resources for evaluating their applicability, limitations and effectiveness.

**Table 1.** Links to the tools covered in this review.

Tools			
Name	Tool #	Link	Description
AlphaFold-Multimer	Tool 1	https://github.com/google-deepmind/alphafold	Model
		https://github.com/sokrypton/ColabFold	ColabFold for using AlphaFold-Multimer
ESM2 and ESMFold	Tool 2	https://github.com/facebookresearch/esm	Model
ESM Atlas		https://esmatlas.com/	Explore the ESMFold predicted structures
ESMFold		https://github.com/sokrypton/ColabFold	ColabFold for using ESMFold
Foldseek	Tool 3	https://github.com/steineggerlab/foldseek	Source code
		https://search.foldseek.com/	Webserver
ProstT5	Tool 4	https://github.com/mheinzinger/ProstT5	Model
Taxometer	Tool 5	https://github.com/RasmussenLab/vamb	Source code. The VAMB package includes Taxometer
SemiBin/2	Tool 6	https://github.com/BigDataBiology/SemiBin	Source code
ProtENN/2	Tool 7	https://github.com/google-research/google-research/tree/master/protenn	Model
ProtNLM	Tool 8	https://github.com/google-research/google-research/tree/master/protnlm	Model
SmORFinder	Tool 9	https://github.com/bhattlab/SmORFinder	Model
GeNLP	Tool 10	https://github.com/burstein-lab/genomic-nlp	Model
		http://gnlp.bursteinlab.org/	Webserver
gLM	Tool 11	https://github.com/y-hwang/gLM	Model
LookingGlass	Tool 12	https://github.com/ahoarfrost/LookingGlass/	Model
Additional tools			
Name	Related to	Link	Description
Uni-Fold	Tool 1 – AlphaFold-Multimer	https://github.com/dptech-corp/Uni-Fold	Faster than AF2 and AlphaFold-Multimer
LucaProt	Tool 2 – ESM2	https://github.com/alibaba/LucaProt	Viral protein classification using ESM2 as component
Foldseek-clusters	Tool 3 – Foldseek	https://cluster.foldseek.com/	Explore the AFDB clusters by Foldseek-clusters
Foldtree	Tool 3 – Foldseek	https://github.com/DessimozLab/fold_tree	Structure based NJ phylogeny
3DiPhy	Tool 3 – Foldseek	https://github.com/nmatzke/3diphy	Structure based ML phylogeny
Foldseek-Multimer	Tool 3 – Foldseek	https://github.com/steineggerlab/foldseek	Multimer extension of Foldseek, source code
		https://search.foldseek.com/multimer	Multimer extension of Foldseek, webserver
Phold	Tool 4 – ProstT5	https://github.com/gbouras13/phold	Bacteriophage annotation using ProstT5 as component
TaxVAMB	Tool 6 – SemiBin/2	https://github.com/RasmussenLab/vamb	Source code. The VAMB package includes TaxVAMB

**Table 2.** Databases used for training the tools in this review.

Name	Used by	Used version	Data type	Metagenomic database?	# entries in version	Comment about the use by the DL-based tool
AFDB	Tool 4 – ProstT5	2024	AA-seqs and their 3D structures as predicted by AF2	Mainly not. AA-seqs originate from UniProt.	214 million predicted protein structures	17 million high-quality, non-redundant and diverse predictions from the AFDB were converted to 3Di tokens by Foldseek and used together with their AA-seqs for training.
KEGG Orthology	Tool 10 – GeNLP	May 14, 2021	Functional orthologs	No. Derived from full genomes.	24 307 KOs consisting of 17 107 806 proteins	KO proteins were further clustered and from each cluster an HMM was computed, yielding 63 234 KO-HMMs, which were used for labeling proteins on contigs.
GTDB	Tool 12 – LookingGlass	Release 89.0	Taxonomic annotation of prokaryotic genes in genomes	Yes. Contains both MAGs and isolates.	145 904 genomes organized in 24 706 species clusters	Each of the 23 458 bacterial and 1248 archaeal genomes was split into read-length chunks.
MGnify	Tool 10 – GeNLP	March 14, 2020	Microbiome data automatically archived and annotated	Yes. Contains various types of data, including amplicon and metagenomic data.	January 2025: 40 094 metagenomes; 481 714 genomes in 12 MAG catalogues	Downloaded all genomes and metagenomic assemblies, excluding those of Metazoa, Fungi, and Viridiplantae, totalling 596 338 genomes and 22 923 metagenomes (with NCBI WGS).
	Tool 11 – gLM	Release 2022-05-06				Used contigs of at least 15 genes, splitting longer ones to chunks of at most 30 genes, resulting in a total of 7 324 684 contigs.
NCBI WGS	Tool 10 – GeNLP	March 14, 2020	Assemblies of incomplete genomes or chromosomes by whole genome shotgun	No.	January 2025: 3 044 366 projects	Downloaded all genomes and metagenomic assemblies, excluding those of Metazoa, Fungi, and Viridiplantae, totalling 596 338 genomes and 22 923 metagenomes (with NCBI MGnify).
PDB	Tool 1 – AF-MM	Max date: 2018-04-30	Experimentally determined protein structures	No.	∼150 000 protein structures	PDB entries were sampled according to the sum of probabilities of their individual chain clusters, which were computed at 40% sequence identity. Sampling blocks for training ensured inclusion of inter-chain regions.
	Tool 2 – ESMFold	Max date: 2020-05-01			∼160 000 protein structures	Only individual chains were used for training. 325 498 chains were clustered at 40% sequence identity, resulting in 25 450 clusters for training.
Pfam	Tool 7 – ProtENN/2	V32.0 and V35.0	HMMs, MSAs and annotations for protein families	Mainly not. Its sequence database pfamseq is based on UniProtKB reference proteomes.	V32.0: 17 929 protein families, each family is associated with 14 545 seed sequences. V35.0: 19 632 protein families	The seed sequences of each Pfam family were split into train, dev and test either randomly or based on sequence similarity. In the random split, the training included ∼1 million sequences (Supp. Table 1 of [85])
SCOPe	Tool 3 – Foldseek	2.01	Hierarchy of single-domain structures	Mainly not.	∼80 000 structures	The domains were clustered at 40% sequence identity, yielding 11 211 non-redundant protein sequences (SCOPe40). The discretization of the 3Di states was learned by splitting SCOPe40 into training (80%) and validation (20%) sets.
UniProt/UniRef	Tool 2 – ESM-2	UniRef50/90 September, 2021	UniProt holds protein sequences and their functional and taxonomic information. UniRefX provides their clustering at X% sequence identity.	Mainly not.	UniProt 2024_06: 254 254 987 proteins	0.5% of the sequences were randomly selected as the validation set. Sequences that matched them with 50% sequence identity were removed from the training set. A minibatch from UniRef50 was sampled for each training update, and each sequence therein was replaced with a randomly selected sequence from the corresponding UniRef90 cluster.
	Tool 1 – AF-MM	UniProt 2020_05				Uniprot taxonomic species labels used for chain pairing input for AF-MM
	Tool 8 – ProtNLM	UniProt 2021_02				The training set was derived from release 2021_02 and yielded 153 502 756 accessions and 231 697 973 sequence-name pairs. The test set was derived from later UniProt entries and consisted of 18 310 527 accessions.

## PROTEIN STRUCTURE PREDICTION AND ANALYSIS

### In this section: AlphaFold-Multimer, ESMFold, Foldseek, ProstT5

In 2021 AlphaFold2 [4] (AF2) was introduced as the first computational method that could accurately predict protein structures directly from protein sequences, with little or no reliance on similarities to known structures. Its revolutionary capabilities alongside the development of easy-to-use interfaces to its models, such as ColabFold ([16,17]) have made it the flagship of DL-based tools, with many thousands of citations and related reviews (e.g. [18–22]).

The original AF2 models were trained to predict single-chain proteins, assuming a single conformation, without any associated ligands or co-factors. Thus, they exhibit limited accuracy in predicting intrinsically disordered regions, protein complexes and protein-ligand interactions (reviewed in [18]). This has motivated and continues to motivate the development of extended and alternative models, beyond AF2 (comprehensively reviewed in [18,23,24]). Most recently, Google DeepMind has announced the release of AlphaFold3 [25], which extends the capabilities of AF2 in various ways, including the modeling of protein-nucleic acid interactions. However, since Google DeepMind released the source code and the model weights under restrictive licenses and since its web server offers limited capabilities, it is not included in this review.

The tools in section I either extend AF2, serve as an alternative to it, or illuminate its results.

### Tool 1: AlphaFold-Multimer

**Purpose**: structure prediction of protein complexes.
**Chosen because**: it is a very important extension to AF2.

#### About

Arguably, the most important extension to the AF2 model is AlphaFold-Multimer [26] because most proteins in nature operate as complexes, rather than monomers [27–29]. AlphaFold-Multimer takes as input the amino-acid (AA) sequences of the chains of the complex, whose structure is desired. First, homologs for each chain are identified based on sequence similarity, then a multiple sequence alignment (MSA) is computed for each chain and its homologs. Next, and unlike AF2, taxonomic information about the sequence homologs of each chain is added in a process called *MSA-pairing* to allow the network to identify the same taxon across chains and utilize this evolutionary information for improved structural inference. In addition, AlphaFold-Multimer ensures that equally-good structural models due to chain symmetries (e.g. when modeling a homodimer) are scored properly. Another important modification in AlphaFold-Multimer relative to AF2 is the inclusion of interface (multi-chain) regions when cropping sequences for training. Other conceptually smaller modifications to the scoring and loss functions, to sequence sampling and to the AF2 neural network architecture are detailed in the original paper [26]. Re-training the modified network using input from the PDB [30] allowed AlphaFold-Multimer to successfully predict homomeric (heteromeric) interfaces in 69% (67%) of the benchmarked cases. This success and the integration of AlphaFold-Multimer models into accessible tools [16,31] have made it possible to use them for predicting the structure of metagenomic protein complexes.

#### Use case: Detect similarity in the twilight zone

van Spanning *et al*. [32] reported the first member of the phylum Actinomycetota (Actinobacteria), which can grow methanotrophically under aerobic conditions. This Actinomycetota species, with the proposed name *Candidatus Mycobacterium methanotrophicum*, was sampled from an acidic biofilm in a metagenomic study of a sulfur cave wall. After assembling its genome, the authors used sequence similarity methods to identify a cluster of candidate subunits of a soluble methane monooxygenase (sMMO), which catalyzes the first reaction in methane oxidation. They then predicted the structure of each Open Reading Frame (ORF) in the cluster as a monomer, using AF2 through ColabFold. This revealed that one of the ORFs was likely a homolog of MmoD, an accessory component of sMMO, despite having only 23% sequence identity to it. When applied to the candidate MmoD and a candidate sMMO α-subunit, AlphaFold-Multimer predicted that the two fold together very similar to a known sMMO, thereby providing further support for the hypothesis of methanotrophy in Actinobacteria.

#### Use case: Bypass Molecular Replacement

Moi *et al*. [33] investigated the origin of sexual reproduction in eukaryotes. Specifically, they identified the first prokaryotic homologs of fusexins, which are required for meiotic sex. The candidate homologs in Archaea were identified by constructing and applying a pipeline of sequence-based tools (e.g. HHblits [34,35]) to several databases, including Metaclust of 1.59 billion clustered proteins from metagenomic and metatranscriptomic studies. Next, an ectodomain region (Fsx1_E_) from one of the selected archaeal candidates was expressed in mammalian cells. Fsx1_E_ formed a homotrimer under certain conditions, which prevented experimental phasing. Moreover, the insufficient sequence identity to solved structures did not allow utilizing *Molecular Replacement* techniques. However, these limitations could be bypassed by applying AlphaFold-Multimer and producing a model of Fsx1. Examining this model allowed the authors to hypothesize that certain ions stabilize the trimeric structure of Fsx1, rather than being required for its formation.

#### Use case: Find potential interactors

Faure *et al*. [36] uncovered mechanisms that Tn7 transposons employ to identify preferred target sites for integration within host genomes. First, Tn7-like loci were identified by a sequence-based search for a signature gene in over a million prokaryotic genomes, deposited in genomic and metagenomic databases. Next, by phylogenetic analysis, the authors identified three candidate target-selector systems in Tn7 transposons. When examining one of the novel target selectors, TnsF, they noticed that it had structural similarity to tyrosine recombinases. However, it was lacking the typical recombinase DNA-binding capacity and therefore they hypothesized it was, instead, interacting with another protein, TniQ. By using AlphaFold-Multimer, they could show that TnsF was predicted to interact with TniQ. AlphaFold-Multimer was also employed to systematically scan for other potential interactors of TniQ.

#### Main limitations

AlphaFold-Multimer poses a heavy computational burden, which means that using a free Colab account is usually limited to the prediction of complexes consisting of up to 1500 AAs [17]. Users who investigate larger complexes or wish to employ AlphaFold-Multimer for a systematic screen of potential interactors will likely need to use paid cloud-based or their own GPU resources. Alternatively, prediction of complex structures can be performed using tools like Uni-Fold [37], which reimplements and accelerates AF2 and AlphaFold-Multimer. In addition, using AlphaFold-Multimer for a systematic screen of potential interactors faces challenges of multiple testing as its false positive rate reaches 20% in some cases [38].

### Tool 2: ESMFold

**Purpose**: structure prediction of proteins.
**Chosen because**: it is an important faster and conceptual alternative to AF2.

#### About

The input for protein structure prediction is an AA sequence. The AF2-based prediction process starts by homolog collection and MSA construction. Based on the MSA, the first network module generates hypotheses about which AAs are in contact with one another, providing them to a second module, which aggregates them to predict the 3D positions of the sequence atoms. The authors of ESMFold [39] adopted a different approach, namely using a language model (LM) as the first module in their architecture, operating directly on a single AA sequence. This protein LM, ESM-2, was trained on over 65 million UniRef [40] sequences, given the task of filling in masked-out AAs based on their context. The model developed attention patterns that correspond to a tertiary structure, indicating the ability of ESM-2 to learn the evolutionary constraints on structures. During inference, the sequence representation by ESM-2 is passed to a folding trunk and a structure module, which operate analogously to the AF2 modules. While not being as accurate as AF2, ESMFold is still a strong predictor, performing well on benchmarks. Its main advantage over AF2 and other methods is its substantial speed-up, decreasing runtimes by 1–2 orders of magnitude. Owing to its speed, ESMFold could generate a valuable resource—the ESM Metagenomic Atlas of over 617 million predicted structures (59% with good confidence) from the metagenomic database MGnify90 [41].

#### Use case: Search the ESM Atlas and predict over 1000 novel encapsulin structures

Kashif-Khan *et al*. [42] constructed and applied a novel bioinformatic pipeline for discovering encapsulins, prokaryotic protein-based organelles, in metagenomic datasets. They first used solved structures of known encapsulins as queries in a structure-based search (using Foldseek, see Tool 3) against confident predictions from the ESM Atlas. In parallel, MGnify sequences annotated with Pfam accessions (using ProtENN, see Tool 7) associated with encapsulins were retrieved from the database. The joint set based both on sequence annotation and structure similarity consisted of 800 000 candidates. Since encapsulins resemble phage proteins, the authors filtered the set in various ways to remove likely phage sequences, retaining ∼1300 putative encapsulins. They then used ESMFold to predict the structures of those which were not included in the ESM Atlas. The predicted structures were compared to one another using DALI [43], to identify clusters of structurally similar encapsulins (see Tool 3, Foldseek, for the same purpose).

#### Use case: Detect highly divergent RNA-dependent RNA-polymerases

Hou *et al*. [44] have integrated ESMFold as a building block in a DL algorithm, LucaProt, designed to classify an input sequence as either a viral RNA-dependent RNA polymerase (RdRP) or not. LucaProt computes two representation matrices from the input: a structural matrix using ESMFold and a sequence matrix, constructed by the extraction of commonly occurring viral and non-viral RdRP subwords. The two matrices are pooled together and passed to other layers to produce a score between 0 and 1, where 0.5 is used as a cutoff for determining if the input is a viral RdRP. After training LucaProt, the authors implemented an additional strategy for identifying viral RdRPs, based on conventional sequence-based clustering. Outperforming the conventional strategy, LucaProt was applied to over 10 000 metatranscriptomes from various environments. LucaProt identified many RNA virus species, belonging to 180 RNA virus supergroups, most of which were estimated to be of novel viral phyla or classes.

#### Main limitations

ESMFold has not been trained on multimers, which is evident in its performance, compared to AlphaFold-Multimer (Supp. Material of [39]). There, ESMFold predicted twice as many complexes incorrectly as AlphaFold-Multimer, and its predictions generally scored worse than AlphaFold-Multimer predictions, even when the prediction was considered correct. Even though ESMFold does not require an MSA as input, it is still implicitly dependent on the homology of its input sequence to the database on which the protein LM, ESM-2, has been trained. This was shown by Rao *et al*. [45] who compared a method for contact prediction based on ESM-1b (the predecessor of ESM-2) to a method which is based on an input MSA. They showed that even though the ESM-1b–based method outperformed the MSA-based method, the performance of both was highly correlated and poorer when the MSA method had shallower MSAs.

### Tool 3: Foldseek

**Purpose**: comparison and search of protein structures.
**Chosen because**: it is essential for processing piles of protein structure predictions.

#### About

The release of AF2 and other protein structure prediction methods has been followed by a major expansion of available protein structures in public databases, such as the AFDB [46] and ESM Atlas (see Tool 2), which contain hundreds of millions of structures. Annotation and exploration of these structures rely on being able to compare them to each other and discover structural similarities through their alignment. Foldseek [47] offers sensitive structural alignment *on par* with gold standard methods, at 4–5 orders of magnitude faster computation time, making it suitable for large-scale analysis. At the heart of the method is its innovative way to encode the tertiary interactions within protein structures, using an alphabet of 20 characters (3Di), thereby representing the structure as a string of characters, on which efficient string-matching techniques can be applied. Specifically, the conformation of the local backbone of each residue is examined alongside the conformation of a close residue in 3D space. The joint descriptors of these conformations are discretized by Foldseek into one of twenty 3Di states using a vector quantized variational autoencoder (VQ-VAE), which was trained on alignments of SCOPe40 domain structures [48]. Additional training was carried out during the development of Foldseek to learn a 3Di substitution matrix. Though Foldseek is not a purely DL-based tool as its core computation involves string comparison algorithms, similar to those developed for MMseqs2 [49], it is included in this review due to its major importance for the analysis of predicted protein structures.

#### Use case: Cluster millions of structures to study protein families and evolution

Barrio-Hernandez *et al*. [50] combined Foldseek with the powerful capabilities of MMseqs2 [49] and Linclust [51] to create a method which can efficiently cluster many millions of structures. In their method, MMseqs2 is used for pre-clustering the structures according to their sequence similarity, reducing the input to a second step, in which a linear-time clustering procedure, similar to Linclust, operates on the 3Di encodings of the structures. By applying this method, ‘*Foldseek cluster*’, to the AFDB, the authors organized its 214 million structures into 2.3 million non-singleton clusters. This allowed them to gain insights about the composition of the AFDB, as they found 96% of its structures belonged to ∼70% clusters, for which annotations were available, while the rest (4%) scattered over ∼30% of the completely unannotated (‘*dark*’) clusters. Furthermore, by examining the taxonomic composition of each cluster, they could identify protein families that are shared by bacteria and humans, suggesting links in very early evolutionary development.

#### Use case: Annotate distant bacteriophages with unprecedented sensitivity

Say *et al*. [52] assembled 227 putative bacteriophage genomes from metagenomic samples in a toxic oil refining environment. After predicting protein-coding genes in these assemblies, they employed sequence-based methods to study them, but these detected similarity to a known protein for only 10% of the putative bacteriophage proteins. Therefore, they predicted the structures of the putative proteins and queried them against the AFDB, using Foldseek. By doing so, the fraction of proteins with similarity to a known protein increased by more than 5-fold. Moreover, combining Foldseek's results with KEGG and GO terms allowed the authors to annotate on average 15% of the proteins in each putative bacteriophage genome, highlighting the importance of structure search for deeper annotation.

#### Use case: Reconstruct structure-based phylogenetic trees

Sussfeld *et al*. [53] identified ancient gene families in a dataset of complete prokaryotic genomes and used them as seeds for searching a metagenomic dataset of over 40 million ORFs, using sequence similarity networks followed by clustering. Phylogenetic trees constructed for some clusters of focus revealed extraordinary divergence as the environmental sequences were deep branching. For two clusters, the authors predicted their protein structures and then used Foldseek to estimate their all-vs.-all average distances (pLDDT). The distance matrix for each of the two clusters was used for computing a dendrogram, using Neighbor-Joining [54] (NJ), indicating high levels of structure divergence. A similar type of analysis was conducted by Han *et al*. [55], who investigated the diversity of reductive dehalogenases in deep-sea cold seeps. They predicted the structures of reductive dehalogenases, used Foldseek to compute their all-vs.-all average distances, and Foldtree [56] to reconstruct a structure-based NJ tree. By combining sequence-based phylogeny and structure-based trees, they could classify cold seep reductive dehalogenases into four distinct groups. Puente-Lelievre *et al*. [57] have taken structural tree inference a step further by computing a 3Di-based rate matrix and using it for maximum likelihood (ML) tree inference. Despite being demonstrated on a single phylogeny, their approach is the first to analyze the 3Di structural alphabet in a full statistical framework.

#### Main limitations

Foldseek is designed for identifying structure similarities between single-chain proteins. This limits its sensitivity for the identification of similarities between multi-chain protein complexes and between disordered regions. Recently, Foldseek has been extended to Foldseek-Multimer [58], which offers a full computational solution for complex-to-complex comparisons. Additionally, Foldseek can only compute pairwise alignments, a limitation which has been solved by FoldMason [59], a progressive aligner for multiple protein structures. Finally, two types of protein regions can be challenging to query with Foldseek. The first are low-complexity structures of mainly helices [60]. Especially when found in non-globular structure contexts, they are encoded by the 3Di alphabet as a repetition of the same character and thus ignored in the prefiltering stage. The second are intrinsically disordered regions, which do not assume a stable structure and therefore the similarities between them are not reflected in the 3Di alphabet.

### Tool 4: ProstT5

**Purpose**: translating AA sequences to structural representations (3Di sequences).
**Chosen because**: it opens the door for ultra-fast exploitation of structural signals.

#### About

The developers of ProstT5 [61] employed Foldseek's (see Tool 3) structure representation using the 3Di alphabet, for extending a protein LM, ProtT5, which was trained on billions of metagenomic proteins. To that end, the weights of a pre-trained ProtT5 were refined in a process, where characters from both the AA sequences and the 3Di representations of millions of AFDB entries were masked-out, training the model to fill them in. The generality of the resulting model, ProstT5, was demonstrated by using it for *transfer learning*. During *transfer learning* its embeddings were successfully provided as input for supervised prediction tasks, like secondary structure prediction and per-residue conservation assessment. The bilingual ProstT5 model can perform various tasks, which require translating from AA sequence to structure representation, and vice versa. These tasks include: (1) protein sequence design (see review [62]) given specific desired structural folds; (2) sensitive pairwise alignment by using ProstT5’s embeddings instead of AA sequences [63]; (3) predicting the structural stability of mutated proteins [64]; and (4) deep-homology search by translating AA sequences to 3Di representations and then applying Foldseek for identifying structural similarities. Of these, the latter task is of most relevance for metagenomic studies, where the computational demand of predicting full structural models for millions of protein sequences is often too grand. When applied to a whole proteome, this ProstT5 translation-based search was shown to be 400–4000 times faster than performing full structural prediction using AF2 followed by Foldseek. This motivated the integration of ProstT5 in Foldseek (release 9 onwards), enabling it to search an input AA sequence, rather than a structure. In addition, ProstT5 is expected to further drive the development of statistical methods for the inference of structural-based phylogenies (see ‘Use case: Reconstruct structure-based phylogenetic trees’).

#### Use case: Develop a tool for annotating metagenomic bacteriophage proteins

The developers of the popular tool Pharokka [65] for bacteriophage genome annotation recently released Phold (https://github.com/gbouras13/phold, no available preprint yet), which they state outperforms Pharokka as it is able to assign more functional annotations to hypothetical phage proteins. Phold first uses ProstT5 to translate input protein sequences to 3Di tokens and then searches them, using Foldseek against a dataset of over a million protein structures predicted from various resources of bacteriophage proteins. By doing so, it gains the high sensitivity of a structure-based search, while being able to process full bacteriophage genomes and metagenomic samples.

#### Main limitations

In case information about the coordinates of the protein atoms in 3D space is required, ProstT5 is not a suitable tool as it can only predict the 3Di representations and not the full structural model. Furthermore, despite being a general model, ProstT5 still underperforms state-of-the-art methods, including its base-model, ProtT5, in tasks like determining residue conservation and sub-cellular localization.

## CONTIG TAXONOMY AND BINNING, PROTEIN CODING GENE CALLING AND FUNCTIONAL ANNOTATION

### In this section: Taxometer, SemiBin/2, ProtENN/2, ProtNLM, smORFinder, GeNLP and gLM, LookingGlass

Several computational processing steps of metagenomic data usually precede gene calling and functional annotation. These include quality control, assembly, taxonomic classification and genomic binning (e.g. [66]), for which DL-based tools have been developed (see, for example, Table 1 of [15]). In the Critical Assessment of Metagenome Interpretation (CAMI) II competition [67], which aimed to identify the best-performing methods for these tasks, none of the winners were DL-based methods, except for VAMB [68], which stood out as one of the fastest genome binners. However, since the CAMI-II competition, several DL-based methods for contig classification and metagenomic binning have been introduced and demonstrated superior performance compared to CAMI-II participants. Consequently, we have chosen to focus Section II on two representative methods for these tasks as well as on the later stages of metagenomic inference.

### Tool 5: Taxometer

**Purpose**: taxonomic classification of assembled contigs.
**Chosen because**: it builds upon existing classifiers and improves their performance.

#### About

Traditional taxonomic classification of contigs is often performed by searching for sequence matches in taxonomically annotated databases. In metagenomic binning, contigs that belong to the same metagenome-assembled genome (MAG) are commonly identified by their similar tetramer frequencies (TNFs) and coverage. Since contigs of the same MAG logically belong to the same taxon, this relationship can inform taxonomic classification. Taxometer [69] classifies contigs from one or multiple samples using a DNN that combines TNFs and coverages with taxonomic labels from existing classifiers. It first runs a base classifier on all input contigs and then establishes the taxonomic tree of the predicted labels. Taxometer encodes the TNFs and coverages through four fully connected layers, producing a vector of logit values that represent the contig's propensities of belonging to each species. From this vector the full model output, i.e. the probabilities of the contig to belong to each tree node, is computed. This computation uses a softmax function for the species and a recursive summation of descendent probabilities for the internal nodes. Taxometer is trained on-the-fly with each input and is not designed to be transferred or generalized between datasets. During training, the loss function is computed between the ‘true’ path on the tree, as determined by the base classifier, and the full model output, scoring precision at all taxonomic ranks simultaneously. Using various CAMI-II benchmarks, Taxometer was shown to improve the results of four base classifiers: MMseqs2-taxonomy [70], Metabuli [71], Kraken2 [72] and Centrifuge [73]. However, the levels to which Taxometer increased the fraction of correctly classified contigs and removed wrong labels varied between base classifiers and benchmarks: better on the gastrointestinal and marine datasets than on rhizosphere.

#### Use case: Improve metagenomic binning

At the time of this writing (Dec. 2024), there are only two citations of Taxometer. One of these citations is by a newly-developed tool by the same group: TaxVAMB [74], which utilizes Taxometer to improve metagenomic binning (see Tool 6).

#### Main limitations

Taxometer was shown to perform as well as its base predictor or better. However, if base classifier A outperforms B, it is likely that base classifier A also outperforms B, even if B is combined with Taxometer (see Fig. 2 of [69]). This means the user still needs to carefully consider the choice of a base classifier. In the same vein, if the base classifier tends more towards classifying wrongly than leaving a contig unclassified (or vice versa), the same tendency is likely to be observed in Taxometer's results. Another limitation is that coverage computation requires mapping reads to contigs and therefore Taxometer cannot be used on previously-assembled contigs when the read information is unavailable, as is the case with metagenomic databases like the Logan assemblies [75].

### Tool 6: SemiBin/2

**Purpose**: genomic binning of metagenomic contigs assembled from short and long reads.
**Chosen because**: it outperforms traditional methods.

#### About

Binning contigs is a critical step for reconstructing MAGs. Traditional *de-novo* binning methods commonly cluster contigs by their TNFs and coverage. In contrast, SemiBin1 [76] introduces a *semi-supervised* Siamese neural network that embeds these input features in 100 dimensions, creating a distance graph on which clustering is performed. The goal of the network is to embed pairs of contigs that belong to different MAGs farther away from each other than pairs that belong to the same MAG. The two Siamese subnetworks share weights and consist of an autoencoder which produces the embedding and a decoder which, during training, reconstructs the input features. Training the network uses two loss functions: an unsupervised one to control the reconstruction of the input by the decoder and a contrastive one, which controls the ability to reconstruct ‘must-link’ and ‘cannot-link’ labels applied to pairs of input contigs. During input preparation, SemiBin1 breaks contigs in half and treats the two halves as a pair with a ‘must-link’ label, meaning they belong to the same MAG. At the same time, SemiBin1 applies the reference-based MMseqs2-taxonomy [70] to the contigs and labels contig pairs as ‘cannot-link’ based on inferred differences in their taxonomy. Using real and simulated benchmarks, including datasets from CAMI-II, SemiBin1 was shown to outperform existing state-of-the-art binners. SemiBin1’s reliance on reference-based taxonomic inference has two main disadvantages: an underrepresentation of contigs that cannot be taxonomically labeled due to their distance from the reference database in ‘cannot-link’ pairs and the computation time of querying the contigs against the database. SemiBin2 [77] overcame these disadvantages by replacing reference-based ‘cannot-link’ pairs with random pairs from the input. This *self-supervised* approach included modifications to the network; mainly the removal of the decoder and using only a contrastive loss function. Like SemiBin1, SemiBin2 uses Infomap-based [78] clustering to handle contigs assembled from short reads and in addition, it uses DBSCAN-based [79] clustering to handle long-read input. SemiBin2 achieved the best results across various benchmarks from CAMI-II as well as real short- and long-read datasets.

#### Use case: Study soil microbial response to combined global change factors (GCs)

Del Río and Rilling [80] analyzed soil samples that were treated with either a single environmental human GC—such as drought or warming—or a random combination of eight GCs. Their goal was to elucidate the MAG compositions of these samples, and thereby reveal the microbial response to the various GCs. The authors binned the contigs assembled from the samples using three tools: SemiBin2, MaxBin2 [81] and Metabat2 [82] and compared the number of obtained bins and their quality using CheckM2 [83]. They found that SemiBin2 with the multi-sample binning strategy provided the best results and used it for subsequent analysis, revealing that combined GCs have a distinctive effect on soil prokaryotic and viral populations.

#### Main limitations

By default, SemiBin1 and SemiBin2 train the network with each run. In case the run consists of a single sample, the training's overhead can be costly. SemiBin1 mitigates this by using pretrained models, learned from multiple samples and shown to give accurate results. SemiBin2 further improved efficiency, saving up to 75% of SemiBin1’s run time on multi-sample runs. Despite these accelerations, SemiBin1 and SemiBin2 remain slower than VAMB (see Supp. Table 4 of [77]). The randomly-selected ‘cannot-link’ pairs by SemiBin2 overcome two main limitations of SemiBin1. However, as noted by the authors, the chance of introducing wrong randomly-selected ‘cannot-link’ pairs increases as the number of MAGs in the input decreases and it is therefore advised to use pretrained models in the case of low complexity samples. Following its publication, two metagenomic binners have been shown to outperform SemiBin2: COMEBin [84] and TaxVAMB [74]. Like SemiBin2, COMEBin adopts a contrastive learning approach. COMEBin generates a multi-view representation of each input contig by randomly extracting fragments from it and computing their TNFs and coverage. These views are combined together by the network and their embeddings are used for clustering the contigs. Despite generally outperforming SemiBin2, COMEBin performed worse on contigs assembled from long-reads (see Supp. Fig. S10 of [84]). Released as a preprint in late October 2024, TaxVAMB employs a *semi-supervised* approach in which, like SemiBin1, a reference-based taxonomic classifier labels the input contigs and Taxometer (see Tool 5) is used to refine the labels. Then, TNFs and coverages computed from the contigs and the refined labels are used to train a bi-modal variational autoencoder, whose embeddings are clustered by *k*-means (short-read) or DBSCAN (long-read). The preprint shows TaxVAMB produces higher quality results than all other binners on various benchmarks. However, the preprint does not include a runtime benchmark and since TaxVAMB uses reference-based classifiers, it may have prolonged runtimes, as SemiBin1.

### Tool 7: ProtENN/2

**Purpose**: associating proteins with Pfam labels.
**Chosen because**: it marks a breakthrough in the ability to perform this core task.

#### About

Associating protein sequences with Pfam families is a common way to annotate them. The developers of ProtENN [85] proposed a DL approach for Pfam association, improving over state-of-the-art methods, which rely on profile hidden Markov models (pHMMs). Their approach consisted of training a set of convolutional neural networks (CNNs), termed ProtCNNs, on a dataset of ∼1 million seed sequences used for constructing the HMM profile of ∼18 000 Pfam protein domain families. The input to each ProtCNN is an AA sequence, which is one-hot encoded and passed through a convolutional ResNet. This results in fixed-length embeddings that are used for predicting the associated Pfam family. The embeddings of the trained ProtCNN are also used by a second classifier, termed ProtREP, which computes their averages, resulting in Pfam family cluster centers. ProtREP can then measure the distance in embedding space from an input sequence to each of the Pfam families in the training set. Based on this distance and a nearest neighbor procedure, ProtREP, like ProtCNN, can associate the input with one of the families in the training set. Unlike ProtCNN, ProtREP can also mark the input as a different (novel) family. The performance of individual ProtCNNs is surpassed by conducting a majority vote among their ensemble of classifications, a strategy termed ‘ProtENN’. After demonstrating that ProtREP and ProtENN generally have lower error rates than pHMM-based classifiers, the authors presented a combined strategy: when the pHMM-based classifier is confident, its prediction is retained, otherwise the ProtENN prediction is used. This strategy added nearly seven million regions from UniProtKB proteomes to Pfam families, an achievement that is comparable with the cumulative effort of applying pHMM-based methods in over a decade.

**See ‘Use case: Search the ESM Atlas and predict over 1000 novel encapsulin structures’**, describing the work of Kashif-Khan *et al*. [42], who were the first to employ ProtENN in a metagenomics study.

#### Use case: Integrate into MGnify 2023

The most recent release of the metagenomic database and annotation resource MGnify [41] included Pfam family and clan annotations produced by ProtENN2 (Bileschi *et al*., no available preprint yet) for each protein residue in the database. According to a white paper released by the authors [86], ProtENN2 builds on principles similar to ProtENN. The main differences between them are that individual residues, not just full sequences, can each be associated with multiple Pfam labels, not just one. The integration of ProtENN2 in MGnify is in combination with pHMM-search by Pfam/HMMER [87], as was proposed for ProtENN. The authors estimate that ProtENN2 contributes a Pfam annotation for an additional 200 million proteins, which cannot be confidently annotated by HMMER.

#### Main limitations

Despite the significant improvement ProtENN/2 offers over existing methods, still many proteins remain unassociated with a Pfam family. Hwang *et al*. [88] report for example that in their dataset of 30 million proteins created from MGnify's genomic contigs, 27.5% could not be associated with any Pfam family using ProtENN.

### Tool 8: ProtNLM

**Purpose**: automatic annotation of protein sequences in English.
**Chosen because**: its inclusion in UniProt and its potential.

#### About

Another effort led by Colwell's group who developed ProtENN/2 (see Tool 7) is the ongoing development of ProtNLM (Gane *et al*., no available preprint yet, draft accessible from https://www.uniprot.org/help/ProtNLM) for annotating protein sequences using meaningful expressions in a natural language (English). An advantage of natural language descriptions is that they are not bound to a limited number of categories, thereby being better equipped for capturing endless variance in protein functions. ProtNLM can be thought of as a translator between protein sequences and English. It is designed as two separately-trained encoder-decoder frameworks where the encoder takes in an AA sequence and the decoder outputs one textual token at a time. The first framework was trained on the Pfam dataset, similarly to ProtENN/2 (see Tool 7). The training aim was to predict for each sequence, several details about its associated Pfam family, including its identifier and a one-line textual description. The second framework was trained to predict the ‘Protein Name’ (PN) field of millions of UniProt proteins based on their sequences. In an impressive effort, the authors first cleaned and standardized as much as possible the vast collection of UniProt textual annotations and sequences. In their draft, they report ProtNLM outperforms pHMM as well as ProtENN in the task of Pfam prediction. For the UniProt task, the authors report that ProtNLM predicted the exact PN field in 57% of the evaluation cases. The rest required further investigation and therefore a sample of them was analyzed by expert curators. Based on this sample, another 35% of the total evaluation cases were estimated to be correct and the rest (8%) remained neither supported nor rejected. Next, ProtNLM was applied to predict the PN field for 56 million uncharacterized UniProt sequences. The authors present further support for ProtNLM's predictions by analyzing the structure of ∼1500 uncharacterized sequences annotated as ‘Cas9’ by ProtNLM. To that end, they predicted the structure of these proteins and used Foldseek (see Tool 3) to search it against the PDB. They found that even low-scoring ProtNLM predictions were hitting Cas9 as their top hit and that among the 100 highest-scoring ProtNLM predictions, the majority were either Cas9 structural homologs (68%) or homologs of similar Cas components (16%). Based on ProtNLM's overall performance, it was selected by the UniProt consortium to provide PN annotations for 49 (of 56) million uncharacterized UniProt sequences.

#### Use case: Examine ProtNLM's hallucinated labels

Durairaj *et al*. [89] investigated the extent to which the AFDB contributed annotations to uncharacterized proteins from UniProt. Focusing on UniRef50 protein cluster representatives (UniProt clustered at 50% sequence identity), they first identified 34% as ‘fully dark’, i.e. cannot be associated with any functional annotation according to a metric they developed. They then leveraged confident structural information to extend functional annotations, thereby illuminating ∼17% of the ‘fully dark’ UniProt50 clusters. Since language models tend to hallucinate labels when prompted with unknown input, the authors hypothesized ProtNLM would predict annotations with more semantic diversity for ‘fully dark’ clusters, compared to ‘fully bright’ ones. Even though both ‘fully dark’ and ‘fully bright’ clusters showed little word variance in their labels, the ‘fully dark’ group was indeed significantly more varied. However, since ProtNLM is still under development, the authors note that a newer version of the software changed many of the hallucinated names to being called uncharacterized proteins.

#### Main limitations

ProtNLM is still a work in progress. While the reported results seem promising, the method has not been fully and stably benchmarked. As demonstrated by the use case, hallucinations can be unstable, confusing and may lead to wrong conclusions.

### Tool 9: smORFinder

**Purpose**: finding of prokaryotic genes shorter than 50 AAs.
**Chosen because**: it focuses on genes with high biological potential.

#### About

SmORFinder [90] is a tool for annotating ORFs shorter than 50 AAs. Its development was motivated by the limited ability of traditional pHMM-based gene finders to reliably detect short ORFs. In a similar strategy to ProtENN (see Tool 7), smORFinder combines pHMMs of known smORF families and DL models that better generalize to unseen families. Its training, validation and test sets were constructed from ∼4500 smORF families identified in a previous study [91] as well as a similar number of spurious ORFs, which could be determined as non-smORFs and served as negative examples. The basic architecture of its DNN consists of three branches for the potentially coding smORF region, for the nucleotides upstream to it and for those downstream from it. The sequence input to each branch is one-hot encoded and passed to three blocks of convolutional, dropout and pooling layers. Then, the three branches are concatenated, continuing to dense layers for producing the output: a classification of the processed fragment. A second proposed DNN slightly varied this architecture, mainly by including a long short-term memory (LSTM) layer in each of the branches. SmORFinder classifies a fragment as a small ORF if a pHMM-based detector or either of the two DNNs produces a highly confident score for it, or if all three methods agree but produce less confident scores. This strategy was shown to produce the best F1 scores.

#### Use case: Mine peptide antibiotics

Torres *et al*. [92] investigated ∼444 000 families of putative small proteins from over 1700 metagenomes obtained from four sites in the human body. These families included over 2.5 million candidates of 15–150 nucleotides in length, which were called with MetaProdigal [93]. Next, smORFinder and AmPep [94], which is designed to detect antimicrobial peptides, were applied to each family representative, resulting in 323 candidates that were scored highly by both tools. Of these, 78 were selected for synthesis and tested for biological activity against 11 pathogenic strains. The authors found that almost 60% of the peptides had antimicrobial activity against at least one pathogen or commensal.

#### Main limitations

The parameters of smORFinder were tuned to prefer precision over sensitivity, thus limiting its recall. In addition, the 4500 smORF families used for training smORFinder can potentially be used for improving traditional pHMM-based methods, like NCBI's Prokaryotic Genome Annotation Pipeline [95], which may reduce the improvement over traditional methods.

### Tools 10 and 11: GeNLP model and web application and gLM

**Purpose**: annotating and organizing prokaryotic proteins.
**Chosen because**: their innovative approach of considering adjacent protein-coding genes on the same contig. These rather new tools (2023/24) have not been put to use in published work yet, but show great potential.

#### About GeNLP

Miller *et al*. [96] used natural language processing (NLP) techniques to predict functional categories for prokaryotic genes based on their genomic context. Their study was the first to show that ‘gene semantics’ can be used for function prediction, even without taking sequences into account. Starting with a corpus of 360 million prokaryotic genes from metagenomic and genomic contigs from NCBI WGS and MGnify, the authors assigned them into gene family clusters based on available KEGG annotations [97], sequence similarity and additional annotations derived from NCBI's *nr* database. This resulted in around half a million unique gene families, of which 20% had KEGG/*nr* annotations and 80% were unannotated. This set served as the vocabulary for training a word2vec neural network, where each contig was treated as a sentence and the family identifier assigned to each of its genes, as a word within the sentence. The word2vec network learned to predict the family identifiers in a window surrounding each gene, based on the family identifier of that gene. Then the word2vec embeddings computed for the annotated families in the vocabulary were used for training a DNN to classify the function of genes, outperforming pHMM-based methods. By applying this network to the unannotated gene families, the authors could reliably annotate ∼10% of the families with a function, spanning over 20 million genes in the corpus. Miller *et al*. have made their pre-trained model available through an online application, GeNLP [98], allowing users to explore functional annotations and the embedding space, based on various types of input, including sequences and KEGG identifiers.

#### Main limitations of GeNLP

By replacing each gene with a gene family identifier, GeNLP effectively treats genes as categorical data and disregards the information borne by their sequences. Furthermore, their word2vec training used a window of 10 surrounding genes, limiting its ability to learn broader gene contexts. Additionally, word2vec is relatively old and often replaced by more advanced models, including transformers [99]. Finally, genomes of non-protist eukaryotes were excluded from the training of GeNLP, potentially limiting its performance on these clades.

#### About gLM

Hwang *et al*. [88] developed gLM (genomic Language Model), a transformer-based LM that considers a continuous representation of genes within a broad genomic context, thereby overcoming the main limitations of GeNLP and other previous methods (see Supp. Table 1 of Hwang *et al*.). Their corpus consisted of ∼7 million prokaryotic contigs or split-contigs of 15–30 genes each obtained from MGnify. As a preparatory step, they associated each gene with a representative protein sequence and then applied the protein LM ESM-2 (see Tool 2) to obtain their protein embeddings. These embeddings within their contig context, appended with gene orientation information serve as the input to the gLM network, which was designed as 19 transformer layers with 10 attention heads per layer. During training, 15% of the genes on each contig were masked and gLM learned to predict their labels: an informative and noise-reduced summary of the embeddings. Since *Escherichia coli* K-12 is a well-annotated bacterium, it was chosen for validating the performance of gLM by removing contigs harboring genes of high sequence similarity to *E. coli* K-12 from the training set. gLM was able to both recover annotation labels as well as learn a confidence metric, indicating the validity of its predictions. Next, the ability of gLM to identify the functional context of genes was demonstrated by the methyl-coenzyme M reductase (MCR) complex, which can carry two reactions and the directionality of its reaction is context-dependent. While the ESM-2 embeddings, which are produced per single protein, could not distinguish between various occurrences of MCR, the gLM-produced embeddings clustered into two groups, corresponding to the direction of the MCR reaction in each instance. The gLM embeddings, unlike the ESM-2 embeddings, could also identify functional clusters like defense systems and biosynthesis. The utility of gLM was then shown for predicting operons, the function of enzymes, and the taxonomy of contigs.

#### Main limitations of gLM

Despite offering ‘higher resolution’ than GeNLP, gLM still focuses only on protein-coding genes, not taking into account any evolutionary information stored in non-coding regions and synonymous mutations. Similarly to GeNLP, the training of gLM was mainly on bacterial, archaeal and viral data, potentially limiting its performance on eukaryotes.

### Tool 12: LookingGlass

**Purpose**: learn a universal language of life on read-length nucleotide sequences.
**Chosen because**: it uses the ‘atomic unit’ of metagenomic data—a short-read sequence.

#### About

Other tools in this review demonstrate the utility of applying DL methods to protein structures and sequences or nucleotide sequences long enough to encode proteins. However, obtaining such sequences usually requires a time-consuming assembly step, which is often incapable of merging all reads. Indeed, large databases like the Sequence Read Archive [100] contain petabases of short reads, most of which still remain unassembled. The authors of LookingGlass [101] set out to learn a universal language of life on read-length nucleotide sequences with the aim of producing a model that can be used for various *transfer learning* tasks. The LookingGlass network consists of three layers of LSTM encoder, followed by a decoder whose goal is to predict the next masked nucleotide from the input sequence. The training, validation and test sets were constructed from disjoint subsets of prokaryotic classes from the GTDB [102]. The genomes in each of the sets were then split into fragments of DNA, whose length followed a realistic distribution of short-read lengths, resulting in 6.6 million fragments in the training set. After training, the learned embeddings were shown to be distinct across different EC numbers and other inference tasks, indicating that the network learned actual biological attributes relevant for protein function. Then, the authors demonstrated how additional learning can be performed on top of LookingGlass, using relatively little data to accurately perform downstream *transfer learning* tasks by training an oxidoreductase classifier that outperforms classification by existing methods.

#### Use case: Develop a predictor of shared protein functionality

Mahlich *et al*. [103] developed a method to explore the repertoires of bacterial functions without relying on reference databases. They first established a dataset of several million unique proteins from thousands of bacterial genomes. Following the ‘Fusion’ protocol they had previously developed [104], they computed a score between each pair of proteins, reflecting their functional similarity based on their sequence identity, as measured by the HFSP method [105]. Next a graph where each node is a protein and two proteins are connected based on their HFSP score was defined to derive >400 000 Fusion clusters of proteins of shared functionality. They then computed the LookingGlass embeddings on the nucleotide sequences of protein pairs marked as belonging to the same Fusion cluster (positive pairs) and to different clusters (negative pairs). These embeddings were used to train a Siamese Neural Network (SNN), which processes two genes at a time and produces a score, reflecting their shared functional similarity. Next, they compared three types of similarity predictions: HFSP-based, SNN-based and structural (based on computed TM-scores [106]). Almost all HFSP-based predictions were of structurally similar proteins, in agreement with the limited sensitivity of sequence-based homology detection. In contrast, SNN-based predictions were also made for pairs without structural similarity, leading the authors to conclude that the SNN-based predictions were orthogonal to sequence and structure signals and, thus, may open the door to investigating remote homology.

#### Main limitations

Despite considering both non-coding and coding regions, LookingGlass is limited in the context from which it learns: short partial genes. As shown by GeNLP (see Tool 10) and gLM (see Tool 11), a broader context of several genes contains important functional and evolutionary information. Trained on prokaryotic data, LookingGlass’ performance may be limited on eukaryotic and viral data.

## DISCUSSION

The advancements in DL have opened up unprecedented capabilities for metagenomic research, impacting nearly every stage of the analysis. Our review focused on 12 tools out of many to concisely demonstrate the cutting-edge advancements and diverse applications of DL in metagenomics, spanning from protein structure prediction to functional annotation. While not performing our own benchmark of these tools, we aimed to describe their performance in the context of other relevant DL- and non–DL-based tools to allow readers to make an informed decision about which tool to use for their analysis.

Looking ahead, several new frontiers are poised to further elevate the impact of DL in metagenomics. Advances in sequencing and assembly methods are likely to produce longer reads and more complete contigs (e.g. [75]), facilitating the training of more accurate and comprehensive models. Additionally, new databases holding high quality predictions produced by current models are likely to drive the development of the next generation of tools, much like the AFDB enabled the development of ProstT5 (see Tool 4), for example. Such databases could include multimer structures (e.g. the FS-MM database [58]), phylogenetic trees inferred from structural signals, and descriptions of proteins in natural language.

Despite the immense promise, it is crucial to acknowledge that DL is not a panacea for all challenges in metagenomics. DL-based tools can vary greatly in their ability to handle metagenomic data from highly diverse environments and organisms. At one end are large language models, such as ESM2 (the foundation of ESMFold) and ProstT5, which were trained on massive datasets but have little out-of-distribution generalizability without retraining. Next are tools relying on homology-based input or specialized training sets (e.g. AlphaFold-Multimer) whose predictions can be improved through utilizing more evolutionary information, even without retraining. Finally, ‘train-on-the-fly’ methods like Taxometer and SemiBin2 learn anew for each input, enabling them to process highly varied data, despite their models not being generalizable.

Another limitation lies in the interpretability of DL models. While these models can uncover patterns and make predictions with high accuracy, understanding the underlying biological mechanisms remains a complex task. Moreover, the substantial computational demand of DL methods, can limit their accessibility and applicability, particularly in resource-limited settings. This demand will only increase with the rise of foundation models (large general-purpose models), highlighting the need for more task-tailored models for metagenomic-scale analysis. Finally, DL is not a golden bullet and does not outperform traditional methods in all cases, as demonstrated by the results of the CAMI II challenge as well as in the limitation sections of the various tools.

In conclusion, DL-based tools will continue to drive new discoveries and deepen our understanding of the microbial world, ultimately translating these insights into practical applications in medicine, environmental science and beyond.
